# Laboring to Conceive: Reducing Barriers to Fertility Care for Same-Sex Mothers Pursuing Parenthood

**DOI:** 10.3390/women2010005

**Published:** 2022-02-23

**Authors:** Caroline E. Richburg, Nina Jackson Levin, Molly B. Moravek

**Affiliations:** 1 Medical School, University of Michigan, Ann Arbor, MI 48109, USA;; 2 Department of Anthropology, School of Social Work, University of Michigan, Ann Arbor, MI 48109, USA;; 3 Michigan Medicine Department of Obstetrics and Gynecology, University of Michigan, Ann Arbor, MI 48109, USA

**Keywords:** infertility, assisted reproductive technology, LGBTQ+, same-sex, lesbian, motherhood, insurance, adoption, sperm donor, parenthood

## Abstract

Infertility clinics and providers in the United States have made efforts to become LGBTQ-inclusive, yet patients in same-sex partnerships continue to face disproportionate barriers to accessing fertility services when pursuing parenthood. This narrative case study of a same-sex couple’s “labor to conceive” illustrates some of the structural barriers to family building that lesbian mothers face when seeking fertility care, including insurance coverage of fertility treatments, federal regulations for sperm donation, and legal definitions of parenthood. Exclusionary medical and legal systems are discussed, as are the informal strategies that this samesex couple utilized to negotiate and circumvent these barriers. A patient-centered model of advocacy that facilitates access to and protection of same-sex partners seeking (in)fertility services is presented. Intervention points at the (1) Logistical and (2) Societal levels are considered with respect to three domains of same-sex reproduction: (A) insurance;(B) sperm donation; (C) legal adoption.

## 1. Introduction

Family-building in the context of infertility has been characterized as an experience of emotional, psychosocial, and financial strain [1–4]. Confronting infertility in heterosexual partnerships has been described as a fraught, “unanticipated life crisis.” Confronting “infertility” (that is, the inability to conceive) in same-sex partnerships produces an entirely new order of difficulty. Professional societies such as the American College of Obstetrics and Gynecologists and the American Society for Reproductive Medicine have established physicians’ ethical duty to provide fertility services without regard for sexual orientation [5,6]. Infertility clinics and providers in the United States (US) have made efforts to become LGBTQ-inclusive, yet patients in same-sex partnerships continue to face disproportionate barriers to accessing fertility services. Despite the increase in same-sex couples who seek fertility services, such couples must navigate medical and legal systems designed for heterosexual reproduction, thereby forcing a “labor to conceive” as they align conditions in effort to render exclusionary systems amenable to their needs [7,8].

The sociolegal challenges to achieving pregnancy in same-sex partnerships are particular to national and regional contexts in which they occur. Especially in cases where socialized medicine rather than privatized medicine is primary, moralized issues of same-sex parenthood may have a different valence. Nevertheless, outside of the US, many nations have addressed and ameliorated societal hurdles. For example, when the Netherlands became the first country to legalize same-sex marriage in 2001, same-sex Dutch couples gained equal rights to their heterosexual peers, including the right to adopt children and obtain legal parenthood [9,10].

In the context of the United States, there is a gap in knowledge about same-sex couples’ barriers to and informal solutions for challenges to family-building. This paper presents a narrative single case study that sheds light on one such “labor to conceive” by a lesbian couple navigating barriers to family building. This account tells the story of Kate and Jess (pseudonyms), a cisgender lesbian couple who live in a politically conservative region in the southeastern United States. Together, Kate and Jess conceived and bore a baby in 2018. Like many heterosexual couples facing infertility, Kate and Jess’s approach to conception involved a pragmatic approach: finances, career considerations, and medical intervention were weighed heavily. However, their story is particularly illustrative of the compounded, structural challenges that lesbian couples face in achieving conception simply by condition of being same-sex. As such, this case aims to poignantly illustrate the systemic medical and legal barriers that lesbian couples interface in the pursuit of assisted reproductive technology (ART). The findings are presented as a narrative account of the medical-legal barriers that Kate and Jess faced in their family-building process with respect to: (A) insuring infertility; (B) sperm donation; and (C) defining parenthood: legal and adoption systems. The discussion considers a patient-centered model of care to advocate for the needs and preferences of same-sex partners seeking (in)fertility services. Intervention points at the (1) logistical and (2) societal levels are considered with respect to three domains of same-sex reproduction: (A) insurance; (B) sperm Donation; (C) legal adoption.

## 2. Materials and Methods

The narrative case study [11] presented here is one of eight case studies examining the pursuit of same-sex motherhood. Participants were recruited through the University of Michigan research study public database recruiting page and through in-person fliers and online forums (such as “Queerception” [12] a subreddit for queer women pursuing motherhood). Inclusion criteria included non-heterosexual identifying individuals and couples who pursued same-sex motherhood using reproductive technologies (regardless of whether a successful pregnancy was achieved). The first author conducted a 75-min semi-structured interview using video-conferencing software in 2018. Informed consent to participate in the study and be recorded was obtained. The interview was audio-recorded and transcribed, and data were de-identified. The first author transcribed and analyzed the data using narrative inquiry and thematic content analyses [13,14]. Participation was voluntary and no incentive was provided. The study was approved by the Institutional Review Board of the University of Michigan (HUM#00150526).

Of note, the authors acknowledge that defining sexual and gender minority identities varies across contexts. Therefore, our use of the term “same-sex couples” refers to cisgender couples of the same sex assigned at birth. Our discussion focuses on cisgender lesbian couples and we use “same-sex motherhood” and “lesbian motherhood” interchangeably to describe their journey to parenthood. We acknowledge that the structural challenges we discuss are particular to the context of the United States, however, we maintain that considering these barriers through a solutions-focused lens applies to same-sex mothers in other regional and political contexts.

## 3. Results

### 3.1. Insuring Infertility

Kate and Jess are a couple in their mid-30 s and 40 s, respectively, living in a southeastern US state. The two met in 2013. A few years prior, Kate reflected on her desire for motherhood when she was diagnosed with cervical cancer and made the decision not to undergo a total hysterectomy in hopes of carrying a pregnancy at a later time. Before the beginning of their fertility journey in 2016, Kate’s partner, Jess, encouraged her to see her gynecologist for concerns about abnormal bleeding—what Kate jokingly called “the infinity period”.

*“I was starting to get all these weird periods and then I was having “the infinity period” where I was bleeding all the time, so finally Jess made me go to the doctor and they did the internal ultrasound. I had a uterine polyp. I’m still paying on that, and I’ll be paying on it for two more years. It was much more expensive than having my baby was—having that polyp removed. And that was why I went to [the fertility specialist]—because I had reached my [annual insurance] deductible. It was like, “well, what else can I get done that my insurance is going to pay for now?” Some of those tests were paid for, but financially, it was going to be so difficult to try to do it. You know, neither of us makes a lot of money. We were trying to [have our baby] in a calendar year for insurance purposes—literally, I was trying to do everything as financially conservatively as possible because we were on such a tight budget that I was literally trying to get everything done. I was trying to plan my pregnancy around my insurance, the busy season at my job, and when I could afford to take off time without messing up the workflow* … *it was super stressful.”*

Having met Kate’s insurance deductible due to the polyp, Kate and Jess chose to pursue motherhood that year to maximize the expenses covered by health insurance. Kate and Jess’s approach to conception was mostly economic, which meant aligning their pursuit of motherhood with the sequencing of their employment and annual health insurance cycle. In this way, the gateway to motherhood for same-sex partners is influenced by economic capital and regulated by insurance companies.

The vast majority of US insurance companies do not cover the cost of fertility treatments regardless of sexual orientation. There are exceptions: 16 states require insurers to either fully cover or offer partial coverage for infertility services. Congressional representatives have taken up this issue in Congress [15–17]. In May 2019, Congresswoman DeLauro (Connecticut) and Senator Booker (New Jersey) introduced bills to the US House and Senate that would mandate insurance coverage of infertility services [16,17]. Unfortunately, policy algorithms like that of Skopos Labs predict a very low probability that these bills are enacted [18,19]. At the time of writing in December 2020, these bills have yet to pass in Congress.

Insurance coverage in the US is dependent upon categorical definition of the condition in question. Infertility is defined medically and for the purpose of insurance coverage as “not being able to get pregnant after at least one year of unprotected sex” in women aged 35 and younger. In women older than 35, the time period is 6 months [20]. This definition is recognized by medical societies such as the Center for Disease Control [20], professional societies such as the American Society of Reproductive Medicine [21], and legal bodies such as the National Conference of State Legislatures [15]. In recent years, this definition has been criticized for upholding heteronormative biases that implicitly exclude single women and lesbians [22,23]. Lesbian women are not always (and often not) biologically infertile; many are simply not part of a male–female relationship assumed within the definition of medical (in)fertility. Insurance companies typically, though not exclusively, maintain this definition of infertility without regard for sexual orientation. Such a definition poses financial barriers to insurance coverage of ARTs for same-sex couples. Many lesbian women do not have the option of trying to become pregnant for up to 6 months or a year at home before entering the clinical domain. In order to count as “infertile” and therefore qualify for insurance coverage (if offered), lesbian women often undergo 6–12 months of monitored inseminations in a physician’s office. Only after failing to become pregnant during this time can they be categorized as infertile. In spite of these categorical barriers, data show that home inseminations have similar success rates to in-clinic inseminations [24,25]. As a result, many same-sex couples are forced to pay for the first 6–12 months of fertility treatments out-of-pocket, which can cost over $14,000 per cycle [15,26]. In prioritizing physiological diagnoses, ARTs exclude those who may benefit most from their possibilities, yet who deviate from the prototypical physiologically infertile patient.

A similar semantic challenge has recently unfolded in France. Prior to 2021, ART in France was regulated through a biological definition of infertility similar to that of the US [27]. The law incorporated social identity, as well, by specifying “man and woman making up the couple” [27]. This changed in 2021 with the passage of a French law that provides access to and cost-coverage for fertility services for all women under age 43 regardless of relationship status or sexual orientation. With this change in policy, France joined 10 other European Union (EU) countries and the United Kingdom (UK) in providing equal care to same-sex couples [28].

Economic and insurance considerations were salient for Kate and Jess, and pursuing motherhood ultimately resulted from incidentally reaching an insurance threshold. The results of Kate’s fertility assessment were encouraging, and Kate and Jess began exploring options for the acquisition of sperm. A serendipitous alignment of encouraging medical results and the couple’s search for sperm led them away from the clinic and into the next phase of their pursuit of motherhood—home inseminations with a known donor.

### 3.2. Sperm Donation

As a barber, Jess befriended a “handsome gentleman with short hair, so she’d see him every 6 weeks or once a month, pretty often.” He joked in conversation, asking when Jess and Kate would have children and if he could be listed as a potential sperm donor. “Ok, you are the list!” Jess responded, laughing. The donor discussed the possibility with his wife and decided to serve as a donor for Jess and Kate (his own motivations were also tied to personal history of adoption and the desire to pay forward the possibility of family-building). Self-insemination is an established option for lesbian reproduction. As noted in Mamo’s 2007 book, Queering Reproduction, the 1984 edition of newspaper-style feminist publication Our Bodies, Ourselves described self-insemination as “the simplest, least invasive and most widely used of the technologies [of reproduction] … it doesn’t require professional help, and we can do it at home” [29]. The edition described how to predict ovulation through cervical changes and directed women to use needleless hypodermic syringes, eye droppers, or turkey basters to self-inseminate with donor sperm [29].

As such, when it came time to conceive, Kate tracked her ovulation by basal body temperature and home urine ovulation predictor kits. When she determined she was ovulating, the couple invited the donor to their home every other day. He would enter Kate and Jess’s bathroom and provide them with what they described as “a specimen in a designated cup” and then leave. After the donor departed, Kate inserted the specimen. They got pregnant on the second try.

As Kate described, the decision to pursue motherhood with a known donor was deeply tied to Kate and Jess’s socioeconomic reality and their relationship with a willing donor.

“The number one reason that we chose to go with a known donor was expense. It’s just very, very expensive for the insemination—for the actual sperm. It seemed like you need several vials per cycle. Maybe the cheapest we saw was $700 a vial, so you need maybe $1400 a month. Plus, that’s just for the sperm, so then you’re also paying [the costs of the medical procedures.]”

The US Food and Drug Administration (FDA) regulates third-party reproduction (eggs, sperm, or embryos that have been donated by a third person(s), but not other fertility treatments) [30]. The FDA requires known donors to have infectious disease labs drawn at specified sites, which are often expensive. Current regulations require infectious disease testing within 7 days of each sperm donation, and sperm is often quarantined per individual clinic protocols for 6 months so that the donor can be retested for infectious diseases that do not immediately test positive in the blood, such as HIV [30]. Donors must also undergo an invasive questionnaire and physical exam and may be ineligible from donation based on factors including “history of sex with another man in the preceding five years” [31]. Thus, commercial sperm is expensive, and purchasing sperm does not guarantee a successful pregnancy.

By using a known donor and home inseminations, Kate and Jess circumvented fees associated with commercial sperm banks and associated medical expenses incurred through conception in a clinical setting. Jess and Kate intentionally minimized interaction with costly systems. Nevertheless, one system that posed nearly insurmountable impediments was the legal system. Despite the ability to control biomedical expenses through careful selection of technologies, same-sex partners are saddled with additional sets of legal actions over their heterosexual peers in order to claim and protect their families. Such actions include termination of parental rights of a known sperm donor; legal adoption by the non-birthing parent; state-sanctioned home study as a part of the adoption process; and marriage if step-parent adoption is pursued.

### 3.3. Defining Parenthood: Legal and Adoption Systems

Over the course of their pregnancy and early motherhood, Jess and Kate worked with three lawyers to secure parental rights of their child. “Adopt your children”, one lawyer advised, explaining that adoption affords stronger protections than birth certificates. According to the National Center for Lesbian Rights (NCLR) [32], when a legally married heterosexual couple has a child, both parents are presumed to be legal parents and are automatically listed on the birth certificate as such. “Presumed parenthood” [32] applies to married heterosexual couples and stands legally unless genetic fatherhood can be disproven in court. Lesbian couples inherently require a sperm donor to conceive, thus one mother will not have a genetic or biological connection to the child (unless one partner serves as the surrogate for the other partner’s embryo, which is less common). Nevertheless, without the possibility of “presumed parenthood”, the non-genetic mother is extremely vulnerable to a shifting political and legal landscape that renders her parental rights precarious. Navigating these legal complexities often requires costly professional counsel. Kate reflected on this reality in her and Jess’ experience.

*“A big challenge for us was the legal fees and having to jump through hoops because we are same-sex* … *We had to [take legal actions to] protect ourselves, to ensure that Jess has custody of her child. We were very irked by—we had to have [the Department of Social Services] come and do a home [study] so the invasion of our privacy, just the concern that they would find something that they thought was problematic. They didn’t, but they could have, so there was some angst around that, and just the paperwork that we had to get in order was probably the most stressful.”*

Because of legal vulnerabilities, NCLR strongly advises same-sex parents—in bolded and underlined text—to adopt their children. Adoptions are more sound than birth certificates because adoption is adjudicated in court while a birth certificate is simply a legal document completed at the time of birth and can be disputed [32]. Court orders are protected under the Full Faith and Credit Clause of the US Constitution (meaning states uphold each others’ judgements; adoption laws vary by state) [33]. Step-parent adoptions have long-existed in every state for the purpose of heterosexual couples entering second marriages who may have children from a previous marriage [34]. These laws are written in gender-neutral language, which can be applied to legally married couples regardless of sex [35]. When the 2015 US Supreme Court Case Obergefell v. Hodges [36] ruled that same-sex couples have the inalienable human right under the constitution to marry in all 50 states, certain parental rights were incidentally conferred upon married same-sex couples, including the ability to partake in step-parent adoptions. “Second-parent adoptions” were born out of the gay rights movement and do not require marriage as a prerequisite. Both step- and second-parent adoptions have been criticized for requiring same-sex couples to adopt their own children—children often conceived as a couple, intentionally, through emotionally and financially costly fertility treatments [35].

Kate and Jess did not have the option to pursue second-parent adoption, as second-parent adoptions still do not exist in their state of residence, even post-Obergefell. For same-sex couples, step-parent adoptions are often used shortly after birth to provide legal protection for the non-genetic mother when second-parent adoption is not available. As Kate noted, same-sex parents pursuing step-parent adoptions must marry to gain parental rights, regardless of the desire to do so [30].

*“I am actually not a fan of marriage—beginning with my childhood—so I didn’t really want to get married. However, in [our state], they only have step-parent adoption, they do not have second-parent adoption, and what that means is that you have to be married. So, we got married when I was very pregnant because we realized, like, we need to* … *”*

Kate and Jess were forced into a “shotgun wedding” of sorts, after a years-long committed relationship and months of emotional and financial expenditure on purposeful attempts to conceive together. Prescribing that same-sex couples must marry to share legal parenthood over their children forces upon lesbian mothers a particular moral status of familyhood not required of heterosexual parents.

Jess and Kate hired a lawyer to first marry them and then, shortly after their daughter’s birth, to complete a step-parent adoption for Jess. Since they used a known donor, Jess and Kate also had to work with a lawyer to terminate the donor’s parental rights to the child. Though Jess and Kate inseminated at home, most clinics will not accept a known donor without a legal contract. These legal actions are essential to legitimize same-sex families in the eyes of the state, yet they often present hidden expenses and barriers. For this reason, the total cost of parenthood for a same-sex couple is, by all accounts, higher than for heterosexual couples. Even if biomedical costs are minimized by using a known donor and pursuing reproductive technologies outside the biomedical establishment, same-sex couples must still undertake often prohibitive legal fees to ensure legitimacy of their parenthood.

## 4. Discussion

Becoming a mother in a same-sex partnership requires active labor. A lesbian woman’s labor to conceive is riddled with a litany of biomedical, financial, and legal contingencies. These intersecting barriers are upheld by physiological definitions of infertility, insurance coverage policies (and lack thereof), and legal definitions of parenthood.

In recent years, activists and medical practitioners have questioned reductionist physiological definitions of infertility itself and advocated for a definition of “social infertility” [22,23,37]. Social infertility describes infertility not based upon a physiological abnormality, but instead upon relationship status. Activists have advocated for “relational infertility” to be recognized as its own diagnosis [37] and for the current definition of medical infertility to expand to include the socially infertile. Such a classification would allow same-sex couples and others who deviate from the prototypical infertility patient eligibility within a diagnostic code that enables access to insurance coverage, support, and protection by medical and legal structures, similar to the broad and inclusive indications for infertility treatments already being used by many European nations [28,38]. Once conception has been achieved, lesbian women must continue their labor to claim and protect their children legally. Thus, lesbian women in the United States who do not have access to legal counsel due to cost, proximity, education, language proficiency, or other exclusionary factors struggle to establish and uphold legal claims to their parental rights, resulting in ever-growing barriers to achieving motherhood.

### 4.1. Interventions

The case of Kate and Jess demonstrates that the pursuit of lesbian motherhood requires intricate and concerted negotiation of multiple intersecting medical, legal, and social systems that are designed by and for heteronormative reproduction to the disadvantage of same-sex couples and certain individuals. In developing a patient-centered model of care that advocates for the needs and preferences of same-sex partners seeking services, we suggest two points of intervention across three domains. These points of intervention include: (1) logistical and (2) societal. The three domains encompass (A) insurance; (B) sperm donation; and (C) legal adoption (See Table 1). The suggested (1) logistical interventions illustrate a patchwork approach to aid same-sex parents in negotiating existing medical and legal systems that structure reproduction from an a priori heteronormative orientation. Larger scale, longer term interventions to the sociocultural landscape of same-sex parenthood call for fundamental shifts in political and social structures governing reproduction and familyhood, which we describe in our (2) societal interventions. Specifically, we ascribe the act of logistical intervention to personnel within social work, patient navigation, or other relevant medical and psychosocial care professions. As negotiating the pursuit of lesbian motherhood borders biomedical and sociolegal sectors, a key professional with expertise at the intersection of both fields is ideal. In the following, we refer to such personnel as an advocate. This matrix and its recommendations are specific to the US sociolegal context. However, considerations of advocacy and legal protections presented here may extend to other national contexts where same-sex couples are vulnerable or excluded.

#### 4.1.1. Logistical

Initial consults between the intended parents and the advocate should include discussions of (A) state- or company-specific policies regarding insurance coverage of medical infertility care, specifically whether coverage is offered at all and—if coverage is offered—diagnostic criteria for infertility. (B) Questions about and resources for sperm donation may be raised, including discussions about the financial, legal, and social dimensions for both known and commercial sperm donation. If directed donation is chosen, parents-to-be should be counseled on legal implications, including the need to terminate the directed donor’s legal parenthood. Parents-to-be should also be counseled on the psychosocial complexities of sperm donation, as many clinics require consultation with a mental health professional for all third-party reproduction (i.e., reproduction that involves donated egg, sperm, embryos, or surrogates) [39]. Often, consults are required for each party individually and all three parties together. These consultations are frequently not covered by insurance. (C) Development and implementation of a plan for legal adoption in accordance with local laws and regulations should be discussed, as well as the legal fees for each relevant legal action. These discussions may include considerations of marriage, separate medical authorizations, guardianship, advance directives, prenuptial agreements, power of attorney, and any other legal documentation. This domain may include finalizing a marriage and preparing legal paperwork to sign at birth. A home study may be required; such an assessment is carried out by a licensed social worker or caseworker and serves as a measure of state oversight on the “capability and suitability of the prospective family to adopt” [40]. The advocate should assist in planning for a home study if the couple’s state of residence requires it. Preparing for the home study may include a home visit by the advocate in preparation for official evaluation.

If all preparations are in place, birth serves as a finalization point for completing all legal actions. The advocate may assist with finalizing actions related to (A) birth-related insurance billing; (B) finalizing paperwork to terminate known sperm donor parental rights, if applicable; and (C) signing adoption papers at birth. Following the birth, the majority of the “labor to conceive” is completed. The advocate may provide continued support to the couple as they adjust to parenthood, connecting them to local resources for future questions and education related to same-sex parenthood. The advocate may compile all relevant insurance, medical, and legal documents that have arisen throughout the pregnancy and birth and transfer those documents to the parents in a manner that will be amenable to quick reference for posterity.

#### 4.1.2. Societal

Fundamental shifts in the sociolegal landscape of familyhood and US infertility treatment are needed to limit disparate barriers and the additional obligations they impose on same-sex couples and their family building pursuits. With respect to (A) insurance, fertility coverage would be more inclusive and equitable if insurers were mandated to either fully cover or offer partial coverage for infertility services in all 50 states, and “social infertility” were incorporated under this purview. Not only would an expanded definition of infertility benefit same-sex mothers, but it would also produce a curb-cut effect [41] that benefits others seeking ARTs including same-sex male couples [42,43], cancer patients who encounter iatrogenic infertility [44,45], people with disabilities [46,47], transgender people [48,49], non-partnered people (or single parents by choice) [50–52], and others whose parenthood pursuit requires ART. This semantic shift is a long-established practice in European contexts, such as in the Dutch and French cases discussed above [28,38]. In terms of (B) sperm donation, commercial sperm donation should be included in insurance coverage of ART. Expanded coverage of gamete donation would also serve heterosexual infertile couples or non-partnered individuals, transgender individuals, and others who require sperm or egg donation as part of their need for family-building. Regarding (C) legal adoption, moving away from a formal definition of presumed parenthood based on genetic association alone would decrease legal vulnerability for same-sex parents and others who use third-party reproduction. Legislative bodies may redefine or expand the definition of “presumed parenthood” beyond genetic parenthood. Such a redefinition would legally ascribe “presumed parenthood of intent” to individuals who intentionally conceived a child—through concerted financial, medical, legal, and emotional effort—which may or may not include a genetic relationship. This “presumed parenthood of intent” would produce conditions that grant same-sex mothers legal custodianship of their children without requiring the new parents to perform a legal adoption at the moment of birth. “Presumed parenthood of intent” may come in the form of recognition by a legal authority of the intent to parent as indicated by pursuit of fertility treatment, and a sanctioned “statement of intent” during pre-conception counseling, or another yet-to-be-conceived sociolegal practice of establishing and upholding parental intent. These legal actions are not unprecedented. The European Court of Human Rights has called for same- and different-sex couples to have “legal recognition and protection of their relationship” [10]. This call to action has led to a variety of new legal family formats, including “marriage”, “life partnership”, “registered cohabitation”, “domestic partnership”, “civil marriage”, “stable nonmarital partners”, etc. [10]. Importantly, gendered language in these legal definitions may have the unintended consequence of rendering transgender individuals vulnerable. Moreover, when a married couple becomes “same-sex” through a gender change, the European Court of Human Rights does not at this time recognize it a violation of rights if a nation forces the couple out of their legal marriage and into a “registered partnership” [10]. The impact of this change on parental rights is yet-to-be-seen.

In sum, these suggested interventions to the domains of (A) insurance, (B) sperm donation, and (C) legal adoption reflect a need for a cultural shift toward normalizing same-sex parenthood through fertility care in a material sense. Attending to these three domains is merely a fraction of the larger and ongoing project of interrogating reproduction, gender, kinship, and technology through medical and sociolegal structures. The possibility of sex without reproduction and reproduction without sex [53] provokes ongoing debates within ethical, legal, financial, political, and technological inquiry. Expanding discourses at the interface of biological realties and their cultural elaborations [54] not only benefits lesbian mothers, but many other individuals who are forced to negotiate exclusionary techno-politics of medical and legal structures within which assisted reproductive technologies are made possible and accessible.

### 4.2. Limitations

As with any case study, this particular account represents the idiosyncratic experiences of one family. The aim of this study is not to make generalizable assertions or claim statistical significance. Rather, we aim to share an illustrative example of multi-faceted challenges that lesbian women and same-sex couples face in pursuing family-building. Our study is limited in its focus to a lesbian couple. As more couples and individuals from diverse backgrounds and identities seek fertility care (or do not), the challenges and barriers faced by those individuals become increasingly diverse. These identities include but are not limited to: gay men; non-partnered men and women; bisexual individuals; asexual individuals; members of the transgender and genderfluid community; those with disabilities; cancer patients and others who face iatrogenic infertility; those of racial and ethnic minority groups; those of low socioeconomic status; and others whose parenthood pursuit requires ART. This article attends to the lived experiences of same-sex cisgender women. Experiences of “laboring to conceive” for non-binary and transgender people merits further research and calls for expansion of the definition “women’s health” are emerging [55]. More research must be done to understand and problem-solve for the unique experiences and potential barriers faced by these individuals and their families, especially for non-binary and transgender experiences with ART. Cataloguing the experiences of non-binary and transgender parents in the US may reveal and reify further barriers to legal parenthood. Moreover, describing the experiences of transgender couples forced from legal marriage into “registered partnership” may highlight further semantic fractures in the international sociolegal landscape. As reproduction is at the center of familyhood for many individuals, experiences of and challenges implicated within fertility care are as diverse as those who reproduce.

## Figures and Tables

**Table 1.** Recommendations for points of intervention.

Point of Intervention	(A) Insurance	(B) Sperm Donation	(C) Legal Adoption
**Logistical**	Discussion of state- and company-specific insurance policies regarding fertility services.	Financing commercial sperm donation vs. directed donation (known donor). Discussion of legal implications of commercial sperm donation vs. directed donation with regard to parental rights.	Discussion of legal documents, actions, and fees: marriage, separate medical authorization, guardianship, advance directives, other legal documentation, (wills, prenuptial agreements, etc), state-specific adoption regulations. Home study prepared; carried out before birth, if possible. Select adoption mechanism of choice and ensure papers are ready for signatures at birth.
**Societal**	Insurance mandated to fully cover or offer partial coverage for infertility in all fifty states, including “social infertility” [23]. Broader Implications: Mandated infertility coverage and the incorporation of “social infertility” into the purview of coverage across state lines benefits many groups.	Incorporating language about third-party/directed donation reproduction into insurance; cost of commercial sperm donation falls under insurance coverage. Broader Implications: Provides coverage for heterosexual infertile couples, non-partnered, disabled, transgender individuals, and others.	Broaden, formalize, and legitimize the definition of “presumed parenthood” to include “presumed parenthood of intent” i.e., parenthood is presumed to be individuals who intentionally conceived the child together, which may or may not be genetic relations. Broader Implications: Supports heterosexual infertile couples vulnerable to presumed parenthood (if using third-party) and other family formations including step-parents.
